# TropiCODB: A multi-omics resource for supporting biodesign in tropical crops

**DOI:** 10.1016/j.bidere.2025.100003

**Published:** 2025-02-27

**Authors:** Wenbao Dai, Shuang He, Yunqing Luo, Chengjun Zhao, Zhijuan Yang, Junyu Zhang, Qibin Wu, Wenquan Wang, Fei Chen

**Affiliations:** a National Key Laboratory for Tropical Crop Breeding, College of Breeding and Multiplication (Sanya Institute of Breeding and Multiplication), Hainan University, Sanya, 572025, China; b College of Tropical Agriculture and Forestry, Hainan University, Danzhou, 571737, China; c National Key Laboratory for Tropical Crop Breeding, Institute of Tropical Bioscience and Biotechnology, Chinese Academy of Tropical Agricultural Sciences/Sugarcane Research Institute, China

**Keywords:** Tropical crops, Multi-omics database, Molecular breeding, Functional genomics, Biodesign

## Abstract

Tropical crops play a pivotal role in ensuring global food security and driving sustainable agricultural development, particularly in regions with limited resources. However, the complexity of tropical crop genomes and the lack of integrated omics data pose significant challenges to genetic improvement and biodesign research. TropiCODB is a comprehensive multi-omics database designed to address these challenges by integrating high-quality genomic, variome, transcriptomic, metabolomic, and phenotypic datasets for eight economically significant tropical crops, including cassava, sugarcane, and oil palm. Advanced tools such as gene family analysis, miRNA profiling, and precision guideRNA design are seamlessly incorporated, offering unprecedented support for functional genomics and molecular breeding. TropiCODB's user-friendly interface and dynamic visualization capabilities enable researchers to efficiently explore key genetic mechanisms and design targeted breeding strategies. By leveraging innovative technologies and curated datasets, TropiCODB provides a robust foundation for advancing tropical agriculture through precision biodesign.

## 1 Introduction

Tropical crops are critical to global food security, economic development, and ecological sustainability, particularly in regions characterized by high biodiversity and limited agricultural resources [1]. Despite their importance, tropical crops face unique challenges, including complex genomic structures, diverse environmental stressors, and limited research resources compared to major temperate crops [2]. Addressing these challenges requires the development of innovative tools and comprehensive data platforms to support research and breeding efforts [[3], [4], [5], [6]].

The advent of multi-omics technologies has revolutionized biological research by enabling the integration of genomic, transcriptomic, metabolomic, and phenotypic data, thereby providing a holistic understanding of complex biological systems [[7], [8], [9], [10], [11]]. However, the application of these technologies to tropical crops remains limited due to the absence of a centralized and well-structured multi-omics database [12] tailored specifically to their unique characteristics. This gap hampers the ability of researchers to explore genetic mechanisms, identify candidate genes, and design efficient molecular breeding strategies [13].

To fill this gap, we developed TropiCODB, a comprehensive multi-omics database dedicated to tropical crops. TropiCODB integrates high-quality datasets across eight economically significant tropical crops, including cassava, sugarcane, and oil palm, which together represent a broad spectrum of agronomic traits and ecological adaptations. These datasets encompass genome assemblies, transcriptome profiles, metabolic pathways, and phenotypic records, all curated from authoritative public resources and experimental studies. TropiCODB also incorporates a suite of advanced tools, including gene family analysis, miRNA profiling, precision guideRNA design, and interactive visualization modules. These tools enable researchers to efficiently mine data, identify functional genes, and prioritize targets for breeding and genetic engineering.

## 2 Results

### 2.1 Overview of TropiCODB

**Fig. 1:**
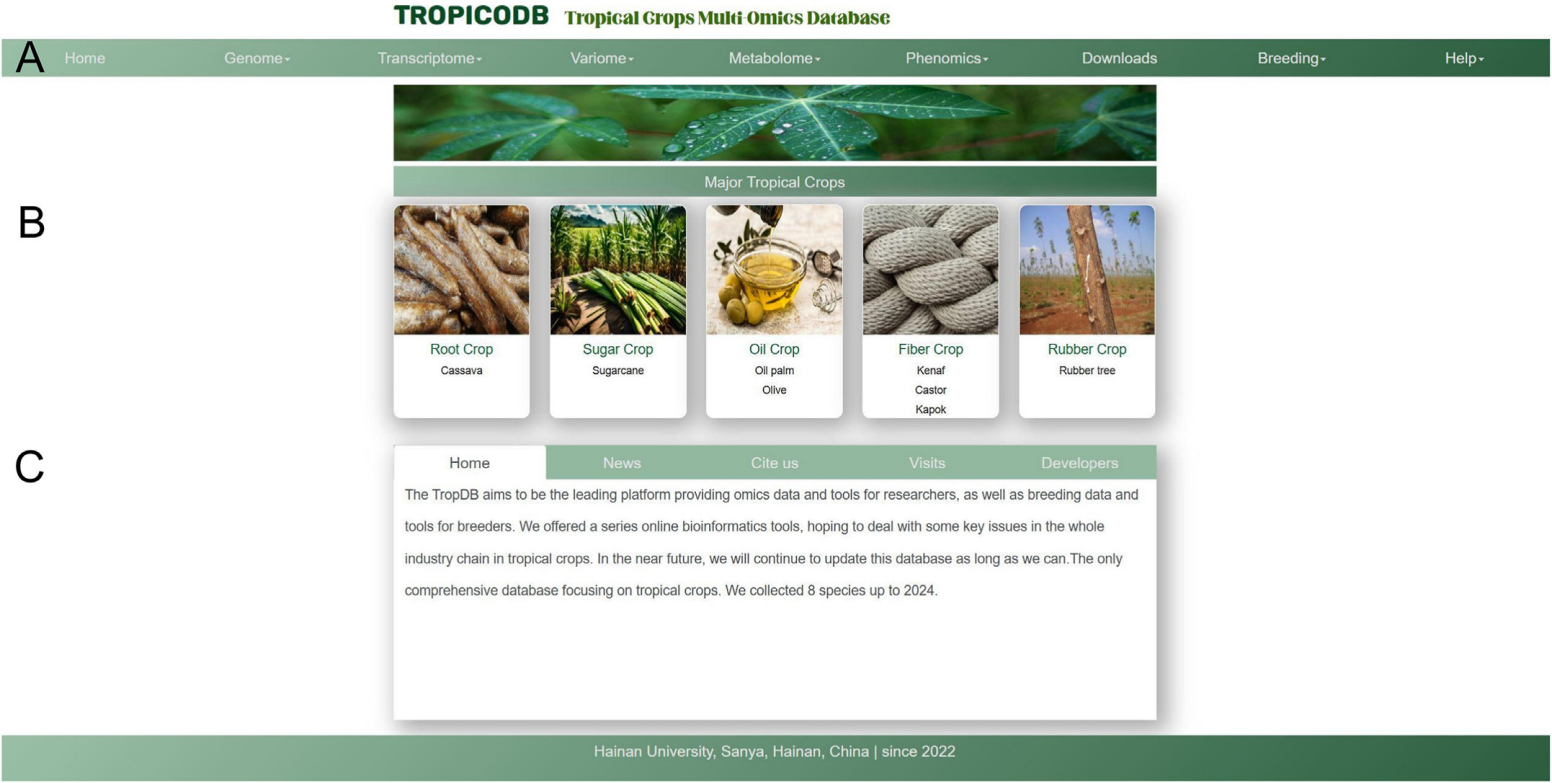
**Overview of the TropiCODB main interface. (A)** Navigation bar: Provides access to various modules, including genomic data, transcriptomic tools, metabolomic insights, and phenotypic data. **(B)** Species classification module: Allows users to browse data and tools specific to the eight tropical crops included in TropiCODB. **(C)** Database introduction: Summarizes the purpose, scope, and features of TropiCODB, highlighting its role as a multi-omics resource for tropical crop research.

**Fig. 2:**
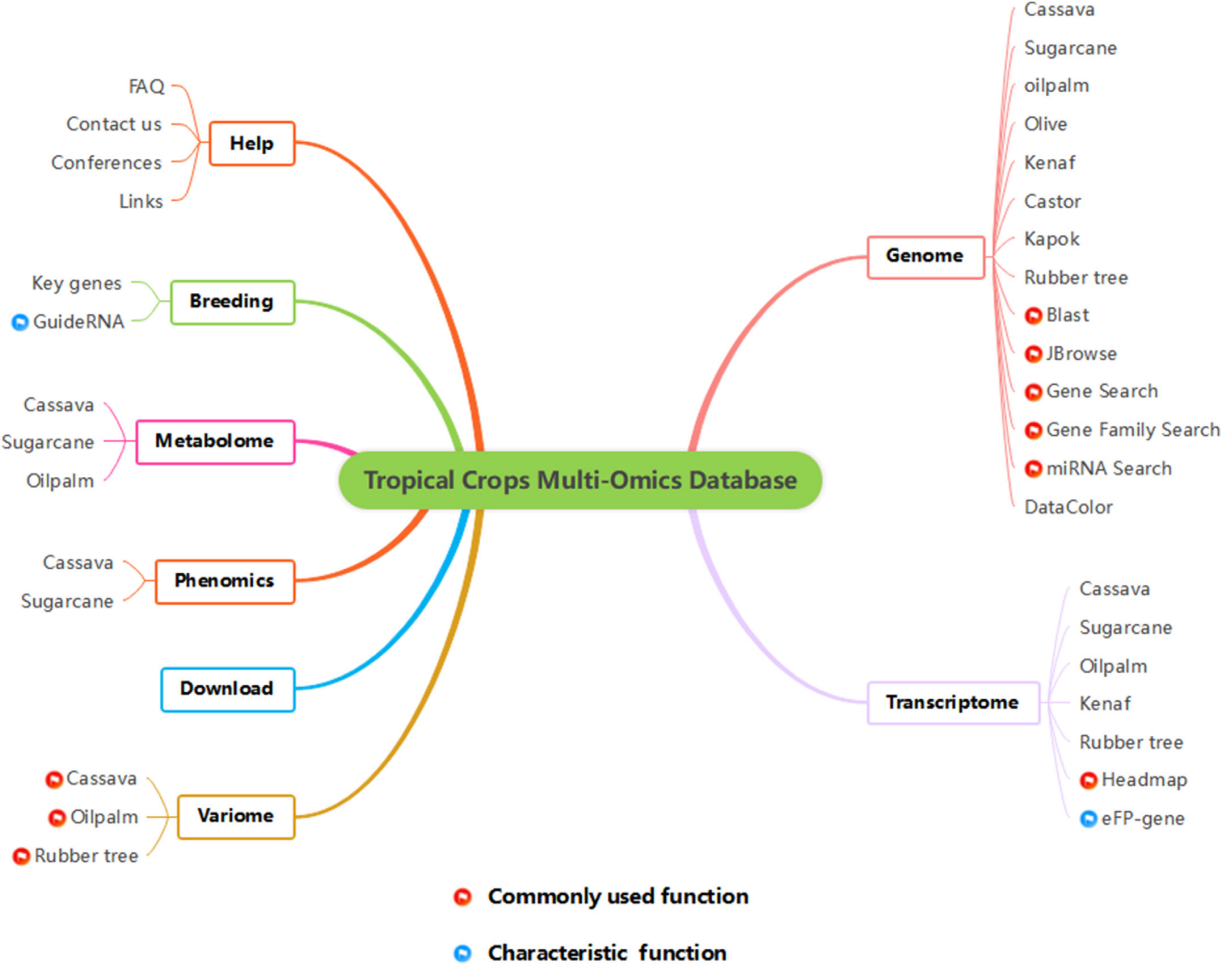
Modular architecture of TropiCODB, showcasing its comprehensive multi-omics resources and unique functional features. TropiCODB is organized into eight primary modules, each addressing critical aspects of tropical crop research, and further subdivided into 34 specialized sub-modules. The design ensures seamless navigation and targeted analysis. Key elements include: **Core Modules:** Focused on genomic, transcriptomic, variomic, metabolomic, and phenotypic data, providing extensive resources for fundamental research and trait discovery. **Specialized Tools:** Highlighted with red flags, these include high-utility functions like BLAST for sequence alignment, Gene Family Search for comparative analyses, and eFP visualization for gene expression profiling. **Unique Functionalities:** Marked with blue flags, TropiCODB offers exclusive features such as precision guideRNA design, species-specific miRNA analysis, and dynamic visualization modules tailored to tropical crops.

TropiCODB is a centralized multi-omics resource that integrates data for five economically significant tropical crops (Fig. 1): food crop cassava (*Manihot esculenta*), sugar crop sugarcane (*Saccharum* spp.), oil crops oil palm (*Elaeis guineensis*) and olive (*Olea europaea*), fiver crops castor bean (*Ricinus communis*), kenaf (*Hibiscus cannabinus*), kapok (*Bombax ceiba* and *Ceiba pentandra*), and rubber crop rubber tree (*Hevea brasiliensis*). This database includes a comprehensive collection of genomic, transcriptomic, metabolomic, and phenotypic data, covering over 20 varieties of these crops. These datasets were sourced from trusted public repositories such as NCBI, NGDC, and Phytozome, and supplemented with experimental data. TropiCODB's design features an intuitive user interface, dynamic visualization tools, and a modular architecture that facilitates efficient data retrieval and analysis (Fig. 2).

### 2.2 Data integration and content

**Genomic Data.** TropiCODB contains high-quality genome assemblies for 20 crop varieties, with varying levels of assembly completeness. For example, cassava includes four genome versions, ranging from early scaffold-level assemblies to the highly complete telomere-to-telomere (T2T) version. Similarly, sugarcane data includes both hybrid cultivar assemblies and wild species references. The genomic data are enriched with functional annotations, including signal peptide predictions, protein domain classifications using Pfamscan, and GO/KEGG annotations. These provide a robust foundation for functional genomic studies.

**Transcriptomic Data.** The database hosts 174 transcriptome samples derived from crops such as cassava, sugarcane, and oil palm. These datasets include expression profiles from different tissues, developmental stages, and stress conditions. Tools for transcript quantification and differential expression analysis are integrated into the platform, enabling users to identify tissue-specific and condition-specific gene regulation patterns.

**Variomics and Population Genomics.** TropiCODB offers comprehensive population data for crops like cassava (573 samples) and oil palm (98 samples). These datasets include SNP and indel variations derived from high-throughput sequencing and processed using state-of-the-art bioinformatics pipelines, such as BWA, GATK, and Picard. Interactive modules allow users to visualize the genomic distribution of variations and prioritize candidate loci for breeding programs.

**Metabolomic Data.** The database includes curated metabolic profiles for key tropical crops. For instance, cassava features 118 annotated compounds, while sugarcane and oil palm include 247 and 1342 compounds, respectively. These datasets enable researchers to study metabolic pathways and correlate them with agronomic traits, such as starch synthesis and lipid biosynthesis.

**Phenotypic Data.** Phenotypic datasets for cassava and sugarcane encompass a wide range of traits, including yield, resistance to biotic/abiotic stress, and quality parameters. For example, cassava phenotypes were sourced from the SeedTracker platform, while sugarcane data were obtained from experimental resources. These data are accessible through interactive tables and visualized via scatter plots and heatmaps.

### 2.3 Functional tools and applications

**Fig. 3:**
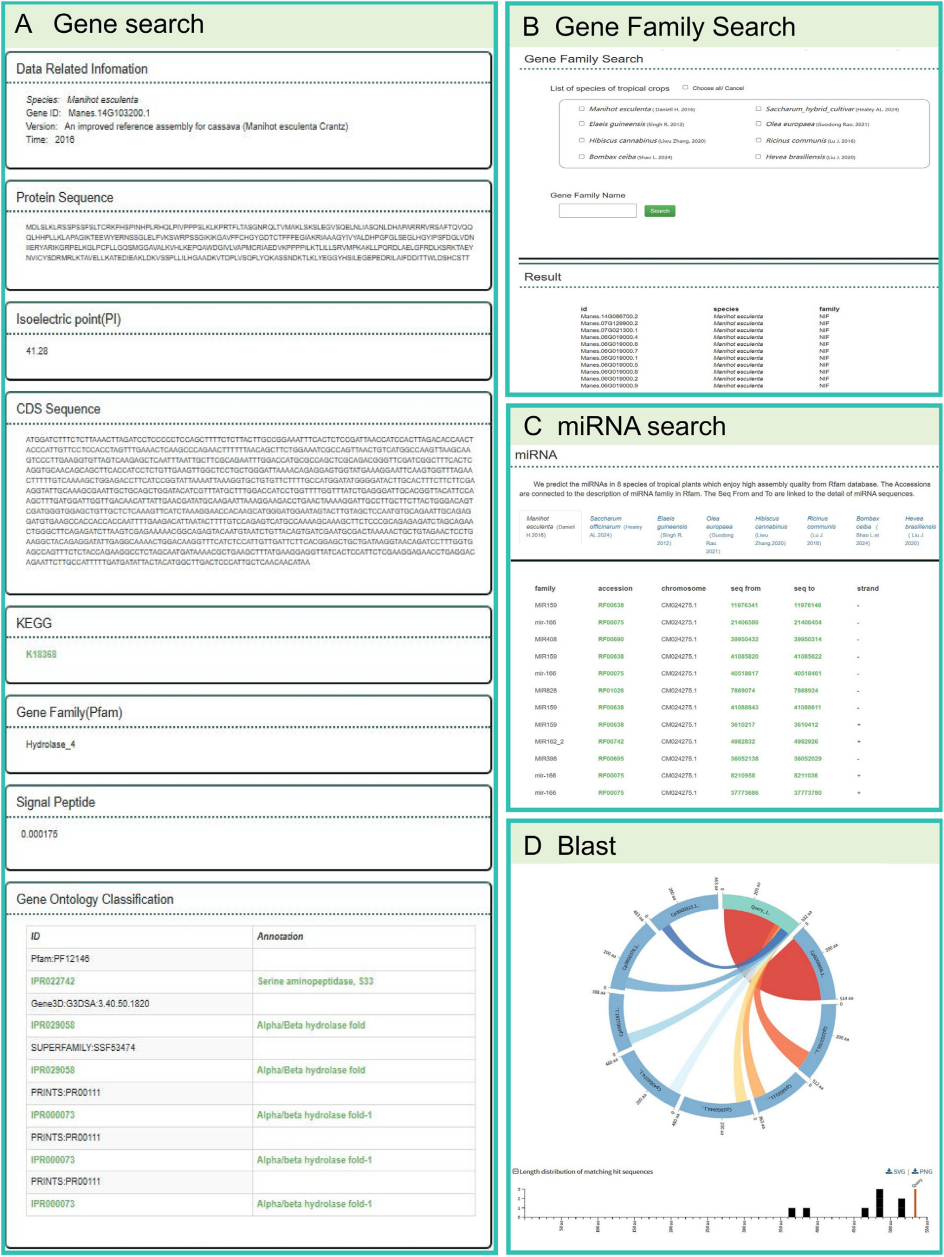
Key analytical tools integrated within TropiCODB for functional genomics and sequence analysis. **(A) Gene Search Tool:** Demonstrates the retrieval of a cassava gene (Manes.12G001200) by its ID, providing detailed annotations, chromosomal location, and associated functional data. This tool facilitates targeted gene exploration and enables researchers to link gene functions with agronomic traits. **(B) Gene Family Search Tool:** Highlights the identification of the nitrogen fixation (NIF) gene family across tropical crops, showcasing phylogenetic relationships and functional conservation. This tool supports comparative genomics and the discovery of evolutionary patterns. **(C) miRNA Analysis:** Displays an example of miRNA profiling in cassava, offering insights into post-transcriptional regulation. The tool allows researchers to explore miRNA targets and regulatory networks critical for crop improvement. **(D) BLAST Tool:** Demonstrates sequence alignment results using a cassava protein sequence as input. This tool supports the identification of homologous sequences across species, aiding in functional predictions and evolutionary studies.

**Gene Search and Family Analysis.** The Gene Search tool (Fig. 3) allows users to query genes by identifiers, annotations, or orthology relationships. For example, users can retrieve detailed information on specific cassava genes, including their chromosomal positions, annotations, and expression profiles. The Gene Family Search tool enables the exploration of conserved gene families, such as the NIF family, across multiple species, facilitating comparative genomics studies.

**miRNA and GuideRNA Design.** TropiCODB integrates miRNA data for cassava and sugarcane, providing insights into post-transcriptional regulation. The guideRNA design tool employs advanced algorithms to generate precise gRNA candidates for CRISPR/Cas-based genome editing. These tools are particularly valuable for targeting genes involved in stress resistance and yield improvement.

**Fig. 4:**
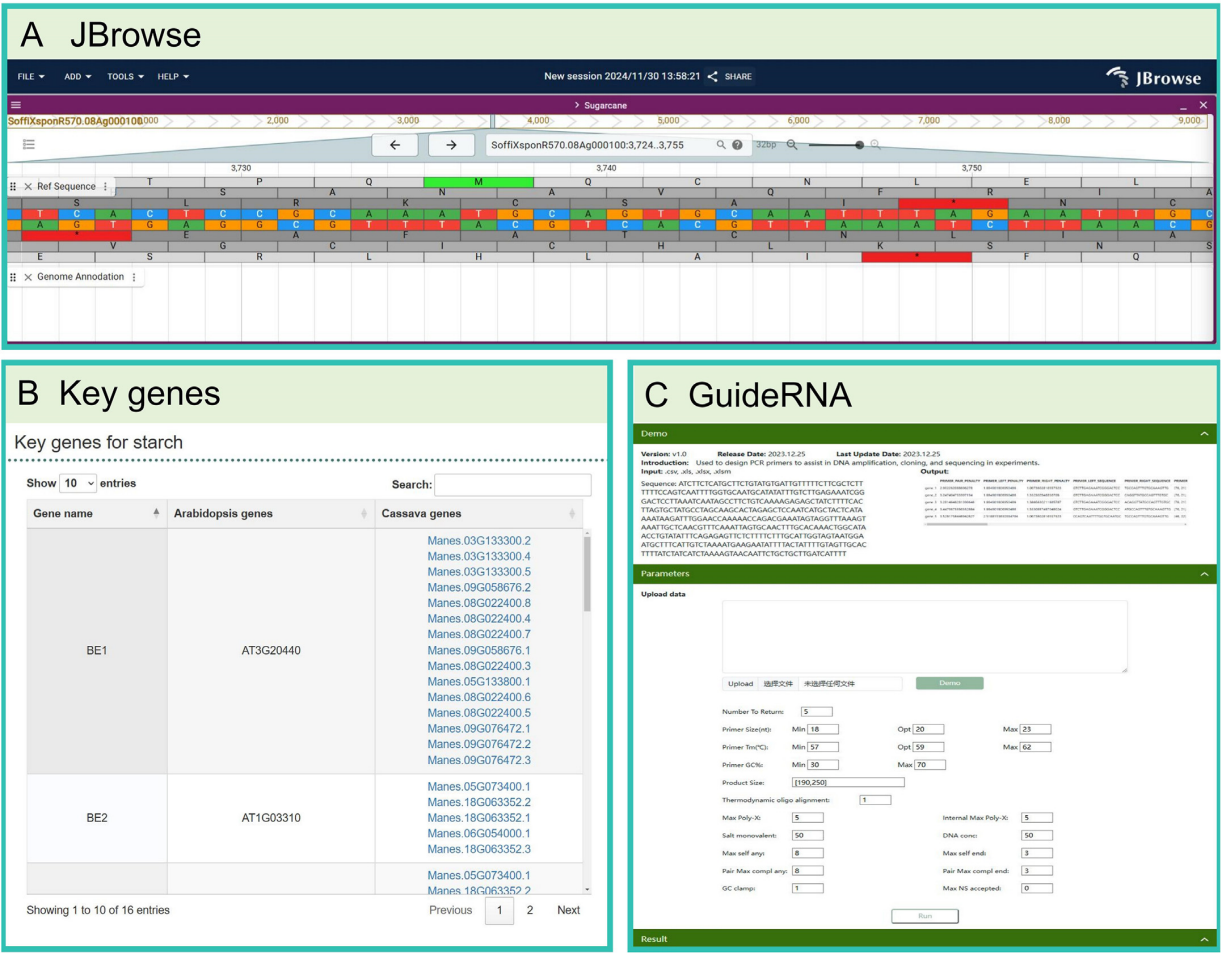
Additional functional tools in the tropical crop database. **(A)** The JBrowse tool displays the chromosome 1 sequence of sugarcane, allowing users to explore detailed genomic information. **(B)** The key gene list shows an example of genes involved in starch biosynthesis and degradation, providing detailed information on relevant functional genes for research purposes. **(C)** The GuideRNA tool provides precise gRNA design results, with an example showing gRNA sequences designed for a specific target region to facilitate gene editing studies.

**Visualization Modules.** Interactive visualization features include chromosomal mapping, heatmap generation, and JBrowse genome viewers (Fig. 4). For example, the JBrowse module allows researchers to visualize specific chromosomal regions of sugarcane, such as key loci associated with sugar biosynthesis. These dynamic visualizations enhance data interpretability and facilitate hypothesis generation.

**Fig. 5:**
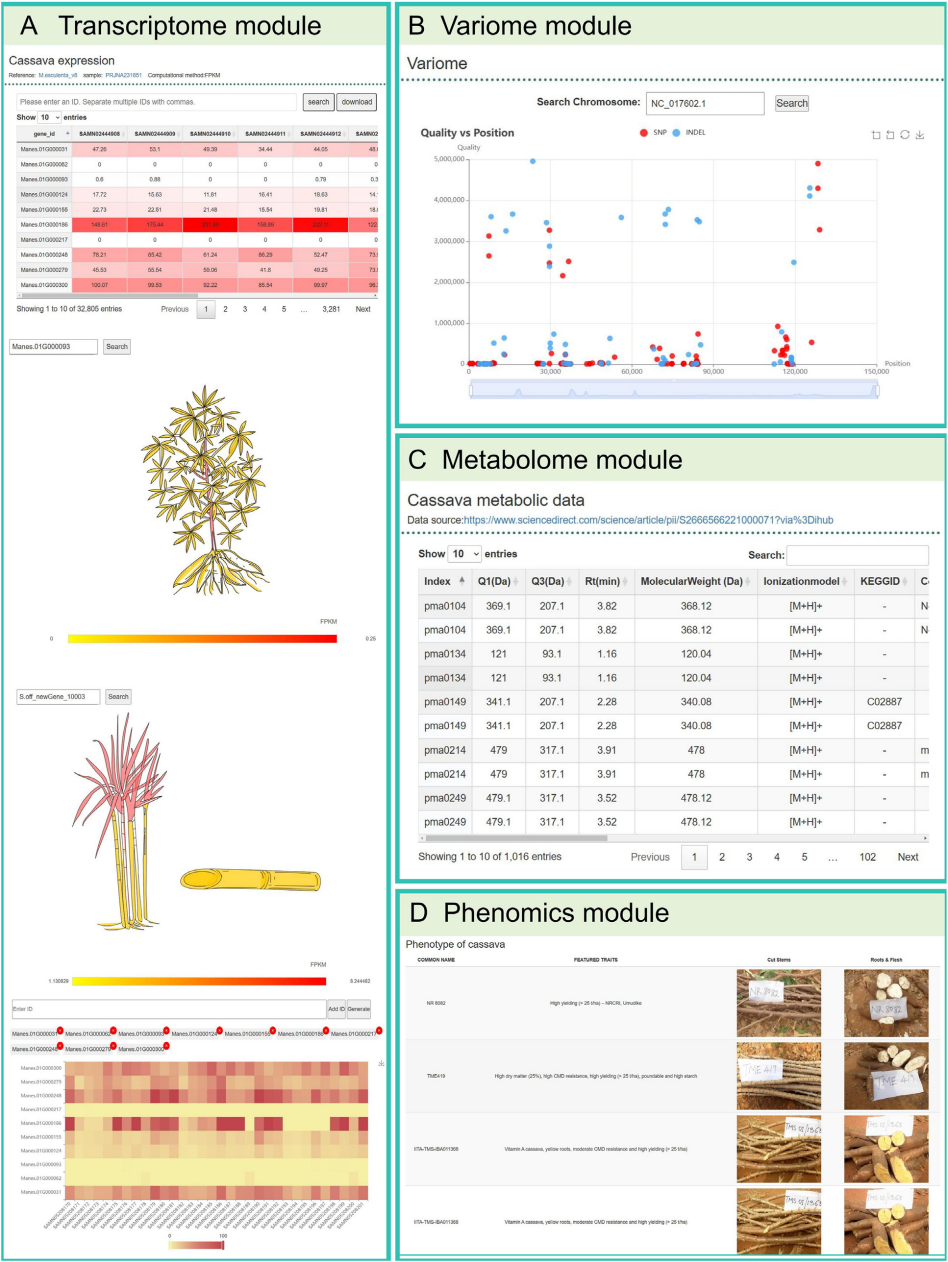
Additional interactive modules in the tropical crop multi-omics database. **(A)** Includes the expression data list, efp-gene visualization tool for cassava and sugarcane, and heatmap generation function for gene expression levels, enabling users to easily examine expression differences and patterns. **(B)** The variantomics module generates scatter plots from variant location information, providing a visual representation of the variant distribution across different samples, aiding genomic variant analysis. **(C)** Cassava metabolic data is presented in a list format, offering detailed information on metabolic products and their variations for in-depth analysis. **(D)** The phenomics module provides a list of cassava phenotypic data as a result example, assisting users in understanding the relationship between phenotypes and genotypes.

**Transcriptome module.** This module within TropiCODB provides a robust platform for exploring gene expression profiles across multiple tissues, developmental stages, and environmental conditions for tropical crops. This module encompasses an extensive collection of RNA-seq datasets, curated from both public repositories and experimental data, covering economically important species such as cassava, sugarcane, and oil palm. One of the standout features of the module is the eFP-gene visualization tool (Fig. 5), which allows users to generate intuitive graphical representations of gene expression patterns. This tool enables researchers to examine tissue-specific expression and identify candidate genes associated with specific traits. For example, cassava's expression profiles reveal root-specific upregulation of starch biosynthesis genes, providing valuable insights for genetic improvement of storage organ yield. The module also integrates heatmap visualization tools, enabling the simultaneous comparison of expression levels across multiple samples and conditions. This feature is particularly useful for identifying differentially expressed genes (DEGs) under stress conditions or during critical developmental stages. Users can input RNA-seq data from various biological replicates, ensuring statistically reliable results. In addition, the transcriptome module includes expression level scatter plots for correlation analysis between genes, facilitating the discovery of co-regulated gene networks. Such analyses are instrumental in unraveling pathways linked to key agronomic traits like drought tolerance, nutrient efficiency, or disease resistance.

### 2.4 Statistical and predictive analytics

TropiCODB incorporates tools for statistical analysis and predictive modeling, such as correlation analyses between phenotypes and genomic markers. It also includes visualization features for population structure and linkage disequilibrium, aiding in the identification of marker-trait associations for breeding purposes.

### 2.5 Use cases and validation

To demonstrate TropiCODB's utility, two case studies were conducted.

#### 2.5.1 Case study 1: Functional Analysis of starch biosynthesis genes in cassava

*Objective:* To identify and analyze key genes involved in starch biosynthesis in cassava using TropiCODB.

**Methodology**

1. **Gene Identification.** Navigate to the "Breeding" section, select "Key Genes," and then choose "Starch Genes" to access a list of genes associated with starch biosynthesis in cassava. Here, you will find the gene Manes.08G022400 as an example.
2. **Gene Search.** Use the "Gene Search" tool to retrieve detailed information on specific genes. For example, searching for the gene ID Manes.08G022400 online. The gene details include the protein sequence, gene sequence, isoelectric point, signal peptide, gene family information, as well as GO terms and KEGG pathway annotations.
3. **Expression Analysis.** Navigate to the "Transcriptome" module, select "Cassava," and then choose "Various Organs" to examine the expression profiles across different tissues. Using the "eFP-gene" tool for visualization, the gene expression in the leaves is depicted with the most intense red color, highlighting the higher expression levels in the leaves.
4. **Sequence Analysis.** Use the "BLAST" tool to compare the sequence of Branching Enzyme 1 (BE1) against other genomes in TropiCODB, allowing you to identify homologous genes in related species.
5. **Functional Annotation.** Review the functional annotations provided in the gene details, including GO terms and KEGG pathways, to understand the biological processes and pathways associated with GBSSI and its role in starch biosynthesis.

**Results**

1. **Gene Identification:** The Branching Enzyme 1 gene (Manes.08G022400) was identified as a key enzyme in amylose biosynthesis.
2. **Expression Analysis:** The eFP-gene tool revealed that Branching Enzyme 1 is predominantly expressed in leaf, aligning with its role in starch accumulation. The results show that Branching Enzyme 1 (BE1) is highly expressed in the leaves, with no detectable expression in other tissues, indicating its essential role in starch biosynthesis.
3. **Sequence Analysis:** BLAST results indicated homologous sequences in related species, suggesting conserved functionality across species.
4. **Functional Annotation:** GO terms associated with Branching Enzyme 1 include "starch biosynthetic process" and "glucan biosynthetic process," confirming its involvement in carbohydrate metabolism.

**Conclusion**

TropiCODB facilitated the comprehensive analysis of the Branching Enzyme 1 gene in cassava, providing insights into its expression patterns, functional annotations, and evolutionary conservation, thereby enhancing our understanding of starch biosynthesis in tropical crops.

#### 2.5.2 Case study 2: SRBP1 gene analyses from cassava

**Methodology**

1. **Gene Search and Functional Analysis.** To investigate the function of the cassava gene *Small RNA-Binding Protein 1* (*SRBP1)* gene Manes.17G101400, a detailed analysis was conducted using TropiCODB. The **Gene Search** tool retrieved comprehensive annotations, indicating that the encoded protein is likely involved in RNA-binding activities. These functions suggest a role in post-transcriptional regulation and immune responses.
2. **BLAST analyses.** The BLAST Comparison tool in TropiCODB was used to align *Manes.17G101400* with the cassava genome, identifying high homology with the *SRBP1* (*Small RNA-Binding Protein 1*) gene.
3. **Gene Expression Analysis.** RNA-seq data from TropiCODB were analyzed to evaluate the expression patterns of Manes.17G101400 in cassava infected with *Xanthomonas.* Expression levels were compared across different time points, focusing on 5 days post-inoculation (dpi).

**Results**

**Gene function and homology**

The BLAST analysis confirmed that Manes.17G101400 shares significant homology with Arabidopsis *SRBP1*. Functionally, *SRBP1* is known to interact with small RNAs, facilitating their stability and regulation under stress conditions. Its role in immune responses and tolerance to cold, salt, and other environmental stresses in Arabidopsis highlights its importance in plant resilience.

**Expression dynamics**

During *Xanthomonas* infection, the expression of Manes.17G101400 in cassava exhibited a notable decrease at 5 days past infection (dpi). This suppression suggests that the gene may be a critical regulator in the early stages of the cassava immune response, possibly involved in modulating post-transcriptional regulatory networks during pathogen attack.

**Conclusion**

The significant homology between cassava Manes.17G101400 and Arabidopsis *SRBP1* implies functional conservation, particularly in RNA-binding and post-transcriptional regulation. *SRBP1*'s well-documented role in enhancing plant immunity by stabilizing small RNAs underlines the potential importance of Manes.17G101400 in cassava's defense mechanisms. The observed downregulation of Manes.17G101400 at 5 dpi may reflect a complex regulatory mechanism in cassava's immune response to *Xanthomonas*. Given *SRBP1*'s role in Arabidopsis, it is plausible that Manes.17G101400 also contributes to fine-tuning RNA dynamics, thereby influencing immune-related pathways and stress adaptation. Further functional studies, such as gene knockouts or overexpression experiments, would be valuable to confirm the regulatory role of *Manes.17G101400* in cassava's response to pathogen infection. This investigation underscores TropiCODB's utility in uncovering homologous genes and providing insights into the genetic and molecular mechanisms underlying plant defense responses.

### 2.6 Summary of database performance

TropiCODB is hosted on a Linux-based LAMP server architecture, ensuring high stability and efficient data retrieval. User feedback indicates that the platform significantly reduces data analysis time while improving the accessibility and usability of multi-omics resources. By integrating advanced functionalities and diverse datasets, TropiCODB sets a new benchmark for multi-omics databases tailored to tropical crop research. It provides essential tools and insights to accelerate functional genomics studies, enabling transformative progress in tropical agriculture.

## 3 Conclusion

TropiCODB represents a transformative resource for advancing multi-omics research and biodesign in tropical crops. By integrating high-quality genomic, transcriptomic, variomic, metabolomic, and phenotypic data for eight economically significant tropical crops, the database provides a centralized platform for exploring genetic mechanisms, identifying candidate genes, and designing molecular breeding strategies. Its user-friendly interface and advanced tools, such as gene family analysis, miRNA profiling, and precision guideRNA design, empower researchers to conduct comprehensive and precise analyses tailored to the unique challenges of tropical agriculture.

The case studies presented demonstrate TropiCODB's potential to address key biological and agronomic questions. From uncovering the genetic basis of starch biosynthesis in cassava to identifying disease-responsive genes in cassava, the database proves invaluable for functional genomics, trait improvement, and plant-disease interaction research. Furthermore, TropiCODB aligns with global efforts to enhance agricultural sustainability and food security, especially in tropical regions where resources are limited, and environmental challenges are significant.

In conclusion, TropiCODB not only bridges critical data gaps in tropical crop research but also establishes a robust foundation for applying biodesign principles to address pressing agricultural issues. By fostering innovation and collaboration, TropiCODB is poised to accelerate advancements in tropical crop science, ultimately contributing to sustainable agricultural development and global food security.

## 4 Materials and methods

**Data Collection and Integration.** TropiCODB integrates multi-omics data for eight tropical crops, including cassava (*M. esculenta*) [14,15], sugarcane (*Saccharum* spp.) [[16], [17], [18]], oil palm (*E. guineensis*) [[19], [20], [21], [22]], Olive(*O*. *europaea*) [23,24], kenaf (*H*. *cannabinus*) [25],castor (*R*. *communis*) [26,27], kapok (*B. ceiba*, *C. pantandra*) [28],and rubber tree (*H. brasiliensis*) [29,30].The data were sourced from trusted public databases such as NCBI, NGDC, and Phytozome, supplemented with experimental datasets. For each crop, the database includes: **Genomic Data:** Genome assemblies with varying levels of completeness, functional annotations such as GO terms and KEGG pathways [31], protein domain predictions via Pfamscan [32], and signal peptide identification using SignalP 6.0 [33]. **Transcriptomic Data:** RNA-seq datasets encompassing diverse developmental stages, tissues, and stress conditions were processed to generate expression profiles [[34], [35], [36]]. Expression data normalization and analysis were conducted using Perl scripts. **Variomic Data:** SNPs and indels were derived from population sequencing data [[37], [38], [39], [40], [41], [42]]. Raw reads were preprocessed with fastp [43], aligned using BWA [44], and variant calling was performed with GATK [45]. Variant annotation and visualization were managed using Samtools [46], Picard, and custom scripts. **Metabolomic Data:** Curated from published literature, the metabolomic data provide insights into key metabolic pathways across species [41,47]. **Phenotypic Data:** Phenotypic traits, including yield and stress tolerance, were obtained from platforms such as SeedTracker (for cassava) and experimental sources for sugarcane.

**Database Construction.** TropiCODB was built using a LAMP (Linux [48], Apache [49], MySQL [48], PHP) architecture to ensure scalability and high performance. Data were stored and managed in MySQL, while dynamic queries and data delivery were implemented using PHP scripts. The front end was designed with HTML, CSS, and JavaScript for an intuitive user interface.

**Functional Tools.** The database incorporates a suite of tools for analysis and visualization, including: **Gene Search and Family Analysis:** Tools for querying genes by identifiers and exploring conserved gene families. **miRNA and GuideRNA Tools:** Features for identifying miRNA-mRNA interactions and designing precise CRISPR/Cas9 guideRNAs. **Visualization Modules:** Interactive charts, heatmaps, and genome browsers, including JBrowse, to enhance data interpretation. **Statistical Analysis Tools:** Modules for correlation analysis, population structure visualization, and linkage disequilibrium mapping.

**Data Visualization and User Interface.** Data visualization was achieved using the ECharts [50] library for interactive plots such as scatter plots and heatmaps. The advantage of ECharts is its high performance, rich interactive features, and flexible configuration, making it suitable for various chart types and large-scale data visualization.Customized JavaScript plugins enhanced table interactivity and enabled seamless navigation between data modules.

## Author contributions

Fei Chen conceived, led, and supervised this study. Wenquan Wang co-supervised the research, reviewed the data. Wenbao Dai designed and supervised the study, performed data analysis, and wrote the manuscript. Shuang He, Zhijuan Yang, Qibin Wu, and Junyu Zhang participated in collecting and analyzing the data. Yunqing Luo assisted with the statistical analysis and provided critical feedback on the manuscript. Chengjun Zhao helped with the data interpretation and contributed to manuscript revision. All authors approved the final MS.

## Data availability

All the codes of this database have been freely available to the public at https://github.com/daiwenba/TropiCODB.

## Declaration of competing interest

The authors declare that they have no known competing financial interests or personal relationships that could have appeared to influence the work reported in this paper.
